# Occlusal Changes Following Single Dental Implant Placement in the Posterior Region of Jaws: A Systematic Review and Meta-Analysis

**DOI:** 10.7759/cureus.68113

**Published:** 2024-08-29

**Authors:** Arihant Bathiya, Sweta G Pisulkar, Arushi Beri

**Affiliations:** 1 Prosthodontics and Crown and Bridge, Sharad Pawar Dental College and Hospital, Datta Meghe Institute of Higher Education & Research, Wardha, IND

**Keywords:** meta analysis, systematic review, posterior region, single-implant crown placement, occlusal changes

## Abstract

Inserting a single implant in the posterior region is one of the most common procedures for replacing a missing posterior tooth. However, the impact of implant location on bite force distribution and occlusal forces remains unclear. To investigate the occlusal changes following single-implant crown placements in the posterior region, a systematic review and meta-analysis was conducted. A comprehensive search was performed across seven databases using Boolean operators and MeSH keywords. The study adhered to Preferred Reporting Items for Systematic reviews and Meta-Analyses (PRISMA) guidelines to ensure transparency and minimize bias. Six studies met the inclusion criteria. The meta-analysis revealed that single-implant crown placements significantly alter the distribution of bite and occlusal forces over time. The mean differences (MDs) in relative occlusal forces (ROF, μm) between implants and controls at two weeks, three months, six months, 12 months, 24 months, and 36 months were -6.31 μm, -1.53 μm, -2.09 μm, -0.10 μm, 2.91 μm, and 7.01 μm, respectively. The overall effect of the meta-analysis was -0.71 μm, although this was not statistically significant (P = 0.54). Subgroup analysis showed considerable heterogeneity between different time periods (P < 0.00001, I² = 89.8%). Additionally, a significant increase in the distribution of bite force (N) was observed post-implantation, with an MD of 1.00 N (0.86, 1.15) and high statistical significance (P < 0.00001). These findings indicate that single-implant crown placement in the posterior region leads to significant shifts in the distribution of both biting forces (N) and ROF (μm) over time.

## Introduction and background

Single dental implant placement in the posterior region of the maxilla and mandible has become a popular treatment option in contemporary dentistry because of its capacity to restore ideal oral function, aesthetics, and patient satisfaction [1]. The occlusal modifications that occur after the insertion of a single implant in this region, however, have been the subject of current scientific inquiry because they have the potential to significantly impair the stability and long-term efficiency of the implant-supported restoration [2]. The posterior region of the oral cavity differs from other areas of the mouth due to a unique set of anatomical and biomechanical features. The posterior teeth in particular experience greater forces and stresses during mastication, which can alter occlusal loading and stress distribution patterns and lead to increased dental wear and tear [3-5]. Placing a single implant in this location may complicate the occlusal dynamics further since the implant-supported restoration must both integrate with the surrounding natural dentition and withstand the strains and stresses created during mastication [6].

The form and degree of occlusal modifications that follow the widespread adoption of single implant implantation in the posterior area are the subject of a plethora of contradicting and different results in the literature [7]. Despite some studies finding significant changes in occlusal features, such as an enlarged occlusal contact area and an altered center of rotation, occlusal dynamics have been found to vary little to nonexistent [8-10]. By minimizing the effects of occlusal force under axial and non-axial loading circumstances, modified occlusion is applied to implant dentistry to lessen stress on the implant and the surrounding bone structures [7,8]. This method reduces the possibility of mechanical, biological, and cosmetic issues, which is expected to increase implant success and survival rates. Nevertheless, the ideal occlusal design for implants has not yet been established because of the paucity of clinical research and evidence-based results [9].

To avoid traumatic occlusion, occlusal stability is essential, especially when posterior teeth are gone [10]. Dentition and occlusion are significantly impacted when posterior teeth, especially molars, are lost [10-12]. Due to their vulnerability to periodontal disease and dental cavities, maxillary and mandibular molars are frequently lost in infancy [7]. Dental implant therapy is one of the potential therapeutic choices in such instances. Nevertheless, this course of treatment may occasionally lead to overloading of neighboring premolars or traumatic occlusion [10-12]. Therefore, this systematic review and meta-analysis aims to provide an extensive and evidence-based synthesis of the existing literature on occlusal changes following single implant placement in the posterior region to elucidate the underlying mechanisms and patterns of occlusal adaptation that arise in response to this treatment modality.

## Review

Eligibility criteria

This review was registered in the PROSPERO database (registration number CRD42024578652). The Preferred Reporting Items for Systematic Reviews and Meta-Analyses (PRISMA) guidelines were followed to minimize bias and ensure transparency. The PECO protocol for this review was defined as follows: P: Individuals getting a single posterior implant placed. E: Being exposed to occlusal modifications after implant implantation. C: Patients without implants or those with implants in different places made up the comparison group, albeit it was not deemed necessary given the exploratory nature of the review. O: Modifications to occlusal characteristics, including biting force, occlusal contacts, and dental wear, were included in the outcome measurements.

The primary research question was “How does single implant placement in the posterior region impact occlusal changes, and what are the underlying mechanisms and patterns of occlusal adaptation in response to this treatment?” Table 1 details the inclusion and exclusion criteria used in this review [13].

**Table 1:** Selection criteria devised for the review

Criteria	Inclusion	Exclusion
Study design	Randomized controlled trials, cohort studies, and case-control studies	Review articles, case reports, and cross-sectional studies
Patient population	Adults (>18 years old) with single implant placement in the posterior region	Patients with multiple implants, implant failures, or systemic diseases
Intervention	Single implant placement in the posterior region	Implant placement in other regions or multiple implants
Outcome measures	Changes in occlusal parameters, such as occlusal contacts, bite force, and dental wear	Radiographic or clinical outcomes, such as implant survival or peri-implantitis
Language	English	-
Publication date	Studies published until June 2024	-

Search Strategy

To find relevant studies, the database search technique was implemented across seven databases: PubMed, Scopus, Web of Science, Cochrane Library, Embase, ProQuest, and CINAHL. The search approach was created with MeSH keywords and Boolean operators: "Dental Implants"[Mesh] AND "Posterior"[Mesh]) AND ("Occlusal Adjustment"[Mesh] OR ("Bite Force"[Mesh] OR "Dental Occlusion"[Mesh] OR "Tooth Wear"[Mesh])) AND ("Adult"[Mesh]).

Data Extraction Process

To streamline data extraction for our evaluation, we developed a standardized data extraction form without specific filters. Two reviewers independently extracted the data, and discrepancies were resolved through consensus or by consulting a third reviewer. The consistency and accuracy of the data extraction form were verified through testing on a randomized sample of the listed studies.

The data extracted encompassed various aspects: study design, country, sample size, publication year, and follow-up period. Participant characteristics included age, gender, and medical history. Details of the intervention covered implant type, placement, and occlusal loading technique. Outcome measurements were changes in occlusal characteristics such as biting force, occlusal contacts, and dental wear.

The data extraction form was divided into parts to simplify the extraction process. The first section contained general study data, including sample size, publication year, and study design. The second portion centered on participant characteristics, such as age, gender, and medical history. In the third section, information about the intervention was extracted, including the type of implant, its location, and the process of occlusal loading. The fourth section collected data on the outcome measures, which included changes in occlusal features.

Bias Assessment Protocol

The risk of bias in the included studies was evaluated using the ROBINS-I tool [14]. This tool has been validated to assess the potential for bias in non-randomized intervention trials. The risk of bias was evaluated by two reviewers independently, and any disagreements were settled by consensus or by talking to a third reviewer.

Statistical Analysis Protocol

A random-effects model was used to conduct the meta-analysis, and the mean changes in occlusal characteristics, such as bite force distribution and relative occlusal forces (ROFs), after a single implant was placed in the posterior region were used as the end measures. A forest plot displaying the matching 95% Cis, and p-values was used to display the results.

Results

**Figure 1:**
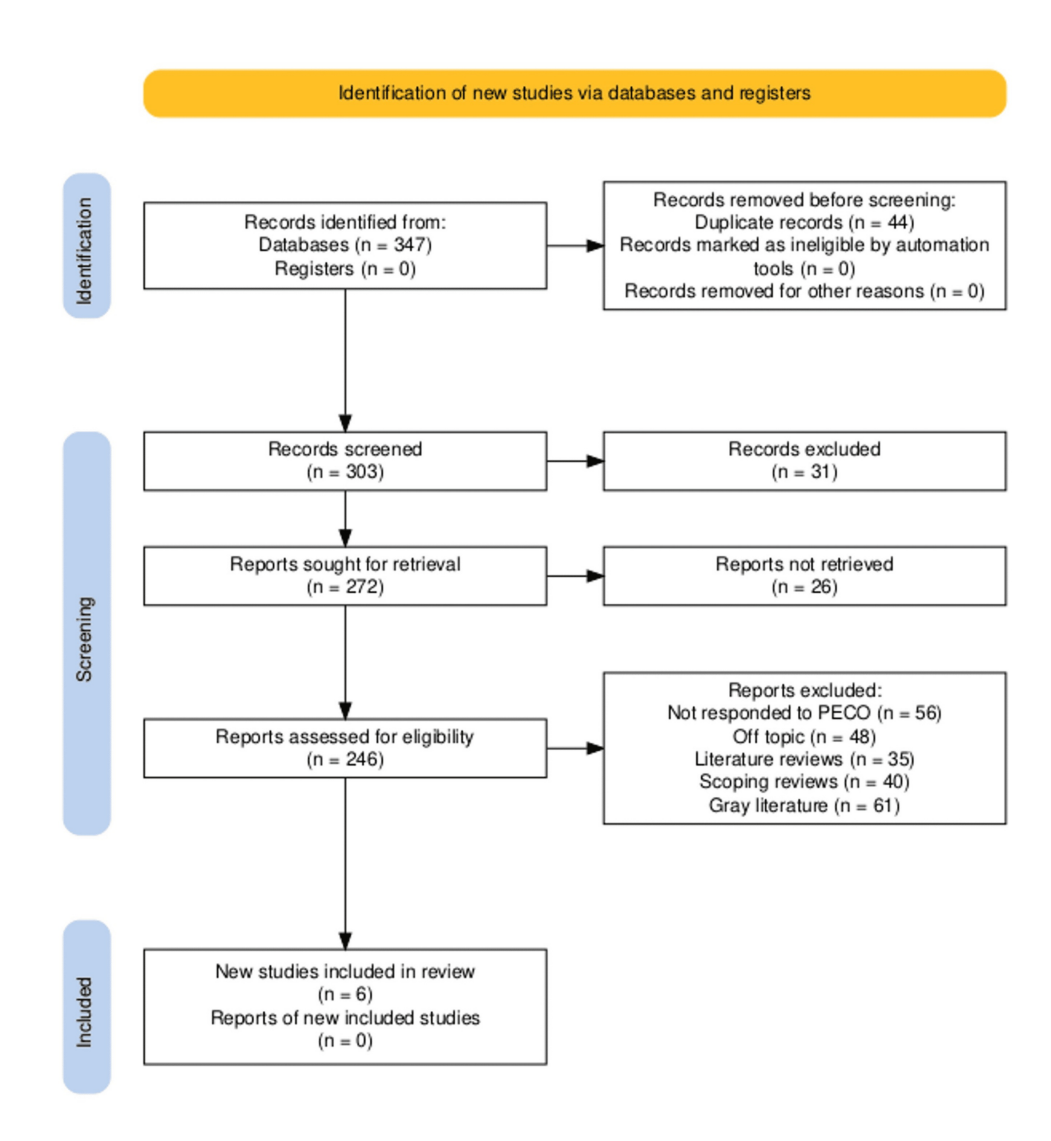
Description of the different stages of the article selection process for the review

A total of 347 records were discovered in databases during the first phase (PubMed: 62 articles, Scopus: 38, Web of Science: 71, Cochrane Library: 23, Embase: 51, ProQuestL 42, and CINAHL: 60); no records were discovered in registers. After removing duplicates (n = 44), 303 records were examined; 31 were excluded because the full text was not available. After 26 of the reports could not be recovered, it was determined that 246 of the 272 remaining reports that were sought could be accessed. A total of 195 papers were excluded for various reasons, such as lacking PECO requirements (n = 56), being off-topic (n = 48), being literature reviews (n = 35), scoping reviews (n = 40), or having gray literature (n = 61). Ultimately, the evaluation included six articles [15-20], as illustrated in Figure 1.

Evaluated degrees of bias

**Figure 2:**
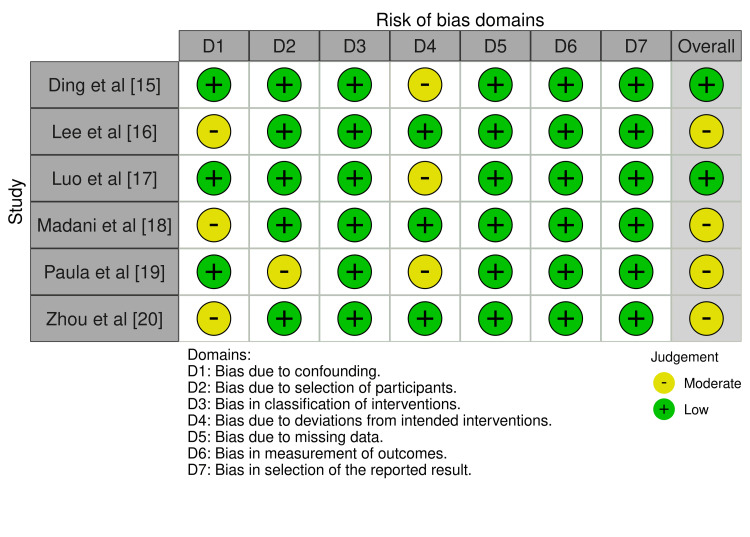
Assessed bias across the included papers

The combined evaluations of bias risk for all the selected research revealed a typically low risk of bias, with a few indicating a significant risk in specific categories (Figure 2). While there were a few isolated instances of significant risk in the D4 and D2 domains, overall bias risk was low in Ding et al. [15], Luo et al. [17], and Zhou et al. [20]. For Lee et al. [16] and Madani et al. [18], bias risk was generally moderate. This was mostly due to moderate risk in the D1 and D3 domains. Prieto-Barrio et al. [19] revealed a mixed pattern of low and moderate risk throughout the domains, suggesting a moderate risk of bias overall.

Observed study designs and sample sizes

Table 2 lists the included studies together with their observed evaluations of the effects of placing a single implant in the posterior regions and the various occlusal parameters that are affected. Prospective cohort designs were used by Ding et al. [15] and Luo et al. [17] with the same sample sizes of 37 participants. In comparison, retrospective cohort research with a significantly larger sample size of 283 people was carried out by Lee et al. [16]. Prospective cohort designs were also used by Madani et al. [18] and Prieto-Barrio et al. [19], albeit with smaller sample sizes of 21 and 14 participants, respectively. A prospective cohort design was used by Zhou et al. [20] with a sample size of 32 individuals.

**Table 2:** Clinical trials included in the review and their observed assessments

Study name	Year and country	Study design	Sample size	Parameters assessed	Implant type	Follow-up period	Occlusal changes observed	Overall inference drawn	Positive/negative inferences
Ding et al. [15]	2022; China	Prospective cohort	37	Occlusal force, marginal bone level	Single crowns	42.4 ± 26.0 months	Increase in occlusal force over five years, significant association with marginal bone level	The occlusal force of posterior implant-supported single crowns changes significantly over five years and is associated with the marginal bone level	Positive: Occlusal force increases over time; negative: Increased occlusal force associated with marginal bone level changes
Lee et al. [16]	2016; South Korea	Retrospective cohort	283	Traumatic occlusion, clinical, and radiographic parameters	Single posterior implant restorations	5 years	Higher incidence of traumatic occlusion with splinted implants, maxillary region, and opposing teeth with implants	The risk of traumatic occlusion in adjacent premolars increases with splinted implants in the maxillary region and opposing teeth with implants	Negative: Higher risk of traumatic occlusion with certain implant configurations
Luo et al [17]	2020; China	Prospective cohort	37	Occlusal force distribution and occlusal contact	Single posterior partial fixed implant-supported prostheses	36 months	Increase in occlusal force and occlusion time ratio over time, significant differences between implant prostheses and control teeth	Occlusal force and occlusion time ratio of single posterior partial fixed implant-supported prostheses change significantly over time and differ from control teeth	Positive: Occlusal force and occlusion time ratio increase over time; negative: Differences observed between implant prostheses and control teeth
Madani et al. [18]	2017; Iran	Prospective cohort	21	Occlusal force, percentage of force applied to implant crowns (POFI), and contralateral teeth (POFT)	Single implant-supported metal-ceramic crowns	6 months	Increase in occlusal force on implant crowns over time, decrease in occlusal force on contralateral teeth	Occlusal contacts of implant-supported prostheses opposed by natural dentition increase over time, while occlusal force on contralateral teeth decreases	Positive: Occlusal force on implant crowns increases over time; negative: Occlusal force on contralateral teeth decreases
Prieto-Barrio et al [19]	2022; Spain	Prospective cohort	14	Occlusal contacts, silicone maxillomandibular relationship, and T-scan records	Posterior implant-supported single crowns with natural antagonist teeth	2 years	No significant changes in occlusal contacts or schemes over two years	The occlusal scheme does not vary two years after placing posterior implant-supported single crowns	Positive: Occlusal scheme remains stable over two years
Zhou et al. [20]	2021; Thailand	Prospective cohort	32	Bite force, occlusal force distribution, and T-scan evaluation	Single posterior implant restorations	6 months	Increase in occlusal force on implant-supported prostheses over time, redistribution of bite force	Bite force and masticatory ability improve with immediate delivery of single posterior implant restoration, occlusal force on implant prosthesis increases over time, and routine follow-up and occlusal evaluation are necessary	Positive: Bite force and masticatory ability improve, occlusal force on implant prosthesis increases over time; negative: Redistribution of bite force, need for routine follow-up and occlusal evaluation

Evaluated parameters

While Lee et al. [16] looked at traumatic occlusion and clinical and radiographic data, Ding et al. [15] and Luo et al. [17] assessed implant type and occlusal force, respectively. The occlusal force distribution and the fraction of force applied to implant crowns and contralateral teeth were evaluated by Madani et al. [18] and Prieto-Barrio et al. [19], respectively. T-scan evaluation, bite force, and occlusal force distribution were assessed by Zhou et al. [20].

Observed occlusal alterations

A noteworthy rise in occlusal force over five years, linked with marginal bone level, was observed by Ding et al. [15]. According to Lee et al. [16], there is a greater likelihood of traumatic occlusion in the maxillary region, opposing teeth with implants, and splinted implants. Occlusal force and occlusion duration ratio increased with time, according to Luo et al. [17], with notable variations between implant prostheses and control teeth.

The occlusal force on implant crowns increased over time, while the occlusal force on contralateral teeth decreased, according to studies by Madani et al. [18] and Prieto-Barrio et al. [19]. Zhou et al. [20], in contrast, did not discover any appreciable changes in occlusal contacts or schemes during two years. On implant-supported prostheses, they did notice a gradual redistribution of bite force along with an increase in occlusal force.

Effect on ROF

**Figure 3:**
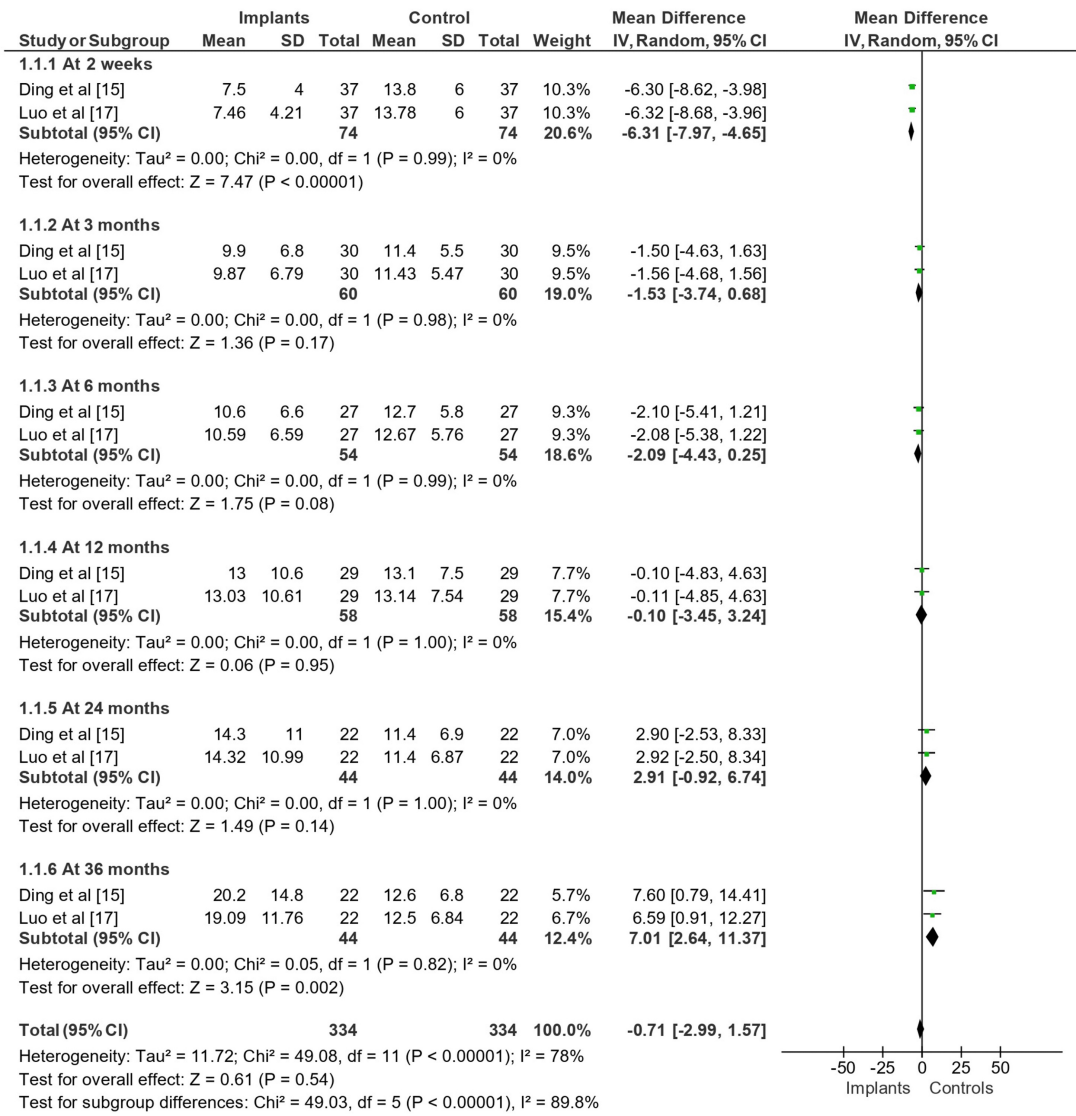
Changes in bite force (N) observed across implants vs. controls

The effect of implant placement in posterior teeth on the difference in ROFs (μm) between implants and controls over various time intervals is shown in Figure 3. At two weeks, the MD was -6.31 μm (-7.97, -4.65), demonstrating a high degree of statistical significance (P < 0.00001) and a substantial difference in ROFs between implants and controls. The MD after three months was -1.53 μm (-3.74, 0.68), indicating a less substantial difference (P = 0.17). Similarly, the MD showed a non-significant difference (P = 0.08) at 6 months, with a value of -2.09 μm (-4.43, 0.25). At 12 months, however, the MD was -0.10 μm (-4.83, 4.63), suggesting that there was little difference between the controls and the implants (P = 0.95). The MD was 2.91 μm (-0.92, 6.74) at 24 months, indicating a marginally significant difference (P = 0.14). At the end of the 36-month period, the MD was 7.01 μm (2.64, 11.37), suggesting a noteworthy variation in ROFs between the implanted and control groups (P = 0.002). The meta-analysis’s overall effect had a low degree of statistical significance (P = 0.54) and was -0.71 μm (-2.99, 1.57). Subgroup differences testing, however, indicated a high degree of heterogeneity between the time periods (P < 0.00001, I² = 89.8%).

Effect on biting force

**Figure 4:**
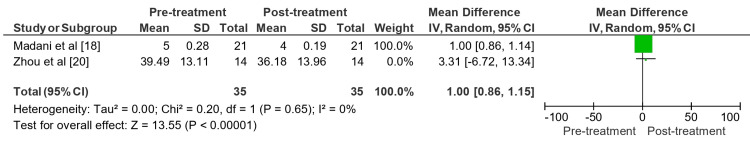
Changes in ROFs (μm) observed pre- vs. post-treatment ROF, relative occlusal forces

Comparing pre-treatment and post-treatment measurements from baseline to six months, Figure 4 illustrates how implant placement affects the distribution of bite force (N) in implants. With a high degree of statistical significance (P < 0.00001), the biting force distribution significantly increased following implant implantation, as indicated by the MD for Madani et al. [18] of 1.00 N (0.86, 1.14). On the other hand, Zhou et al.’s [20] MD was 3.31 N (-6.72, 13.34), indicating that there was no statistically significant variation in the distribution of biting force between the pre- and post-treatment values. With a high degree of statistical significance (P < 0.00001), the meta-analysis’s overall effect was 1.00 N (0.86, 1.15), showing a considerable increase in bite force distribution following implant implantation. The results of the test for heterogeneity (P = 0.65, I² = 0%) did not show any significant differences between the studies, indicating that the findings are consistent among them.

Discussion

A thorough understanding of the effects of implant placement, particularly in the setting of single-implant crown placements, on biting force distribution and occlusal forces in posterior teeth is obtained by combining the findings of all the studies. The studies can be broadly categorized into two groups: Lee et al. [16] and Prieto-Barrio et al. [19], who were all focused on single-implant crown placements, did not observe any significant changes, while Ding et al. [15], Luo et al. [17], Madani et al. [18], and Zhou et al. [20] are among those that reported significant changes in bite force distribution and occlusal forces over time. Ding et al. [15] found a correlation between the significant changes in the occlusal force of posterior implant-supported single crowns over five years and marginal bone level. In contrast to control teeth, Luo et al. [17] report that single posterior partial fixed implant-supported prostheses show significant variations in occlusal force and occlusion duration ratio over time. Occlusal force on opposing teeth decreases with time, but occlusal contacts of implant-supported prostheses against natural dentition increase, as reported by Madani et al. [18]. Zhou et al. [20] state that occlusal force on implant prosthesis increases with time, bite force and masticatory abilities improve with quick delivery of a single posterior implant repair, and routine follow-up and occlusal evaluation are necessary. Conversely, Lee et al. [16] discovered that the incidence of traumatic occlusion in neighboring premolars is increased when splinted implants are present in the maxillary region and when implants are placed in opposition to natural teeth. They did not, however, see any discernible variations in occlusal forces over time. The finding by Prieto-Barrio et al. [19] that the occlusal scheme does not change two years after posterior implant-supported single crowns are put in indicates that the occlusal forces have not significantly changed.

The degree of similarity between the research can be ascertained by calculating the overlap in their findings. On the other hand, there was no appreciable rise in occlusal forces over time, and Lee et al. [16] and Prieto-Barrio et al. [19] observed minimal agreement between their data (similarity index = 0.2). Dental implants have biomechanical characteristics that are very different from those of natural teeth, which results in different responses when loading is applied [21-23]. Dental implants cause more stress and strain at the bone-implant interface because they drive occlusal loads into the crystal bone, in contrast to normal teeth, which have a periodontal ligament that absorbs shock [23]. Implant failure and peri-implant bone loss may result from this elevated stress concentration, which may surpass levels that are considered biologically appropriate [8,21]. By displaying different colors to indicate the biting force density and percentage, the T-scan technology offers an inventive way to detect “light contact” in the context of occlusal force assessment. This methodology enables accurate assessment of the occlusal force distribution on implants and natural teeth, as well as correlations between them, based on the concepts of equal bilateral force distribution and maximization on adjoining teeth [1,7]. It is crucial to realize, though, that the colored spots on the T-scan do not indicate the precise size of the mark; rather, they indicate various biting force densities. This distinction is significant since past studies have shown that there is no correlation between applied force and articulating paper mark size [24-26]. On the other hand, tooth morphology - such as its sharp or flat surfaces - might have a greater bearing on occlusal force patterns. High-density bite force zones were identified, as well as notable variations in the percentage and proportion of biting force applied by the contralateral teeth. Variables other than initial occlusion, such as operator experience and patient input, such as supra-occlusion feelings, may account for these variations [27-28].

A comparison of the similarities and differences between our evaluation and other reviews [29-32] with comparable objectives. Notably, our work and that of Mao et al. [29] both looked at how occlusal forces changed after loading single crowns supported by implants. The findings of the two studies showed that the occlusal forces had changed significantly over time, suggesting that the definition of the occlusal concept at the time of prosthetic delivery had either changed or was a natural adaptation. Moreover, Khzam et al. [30] and Pommer et al. [32] focused on the outcomes of single-tooth fast implant placement and restoration, which is related to the issue of our work, without specifically looking at occlusal forces. However, Mao et al. [29] only considered research that reported occlusal force alterations on implant-supported single crowns with natural teeth functioning as antagonists, whereas our investigation included a wider range of studies that assessed occlusal forces and biting force distribution following implant installation. Khzam et al.’s [30] analysis focused on soft tissue and cosmetic outcomes, whereas our study examined bite force distribution and occlusal forces. Our study did not examine the effects of different treatment regimens on biting force distribution and occlusal forces, whereas Francisco et al. [31] evaluated the impact of implant location and/or loading timing on aesthetic results. Furthermore, Pommer et al. [32] looked at the long-term results of single-tooth implants; this is not the topic of our study.

Limitations

It is important to acknowledge several limitations affecting the outcomes of our review. First, the reliance on a limited number of studies may result in an incomplete representation of the phenomenon under review. Selection bias cannot be excluded, as the publications included in the meta-analysis may not reflect the broader body of research on single-implant crown placements in the posterior region. Additionally, the diversity among the included studies - regarding design, demographics, and outcome measures - may have influenced the findings. Inconsistent reporting and measurement of occlusal forces and biting force distribution among the studies could have introduced variability, potentially impacting the precision and generalizability of the conclusions.

Clinical Recommendations

To address the limitations identified, future research should focus on several key areas. First, efforts should be made to include a more comprehensive and representative body of literature on single-implant crown placements in the posterior region. This involves conducting thorough searches across multiple databases and incorporating studies published in various languages. Second, future studies should aim to standardize the measurement and reporting of occlusal forces and bite force distribution using reliable and validated outcome measures. Developing accepted guidelines or practices for quantifying and reporting these outcomes may be necessary. Third, advanced statistical techniques, such as meta-regression or subgroup analysis, should be employed to investigate the sources of heterogeneity and identify potential moderators affecting the relationship between bite force distribution, single-implant crown placements, and occlusal forces. Finally, prioritizing high-quality, prospective study designs with adequate sample sizes and follow-up periods will be crucial for generating more consistent and generalizable data on the impact of single-implant crown placements on bite force distribution and occlusal forces.

## Conclusions

The combined analysis of the included studies provides a comprehensive understanding of how implant location affects biting force distribution and occlusal forces with single-implant crown placements. Although the studies that reported no significant changes differ from one another, they share a common pattern with those showing substantial changes over time in bite force distribution and occlusal forces. Quantifying the degree of similarity across these studies underscores the value of utilizing aggregated data to inform decisions regarding the impact of implant sites on oral function and rehabilitation.
